# Determination of a DNA repair-related gene signature with potential implications for prognosis and therapeutic response in pancreatic adenocarcinoma

**DOI:** 10.3389/fonc.2022.939891

**Published:** 2022-10-24

**Authors:** Jinzhi Lai, Weijie Chen, Aiyue Zhao, Jingshan Huang

**Affiliations:** ^1^ Department of Oncology, The Second Affiliated Hospital of Fujian Medical University, Quanzhou, China; ^2^ Department of Surgical Oncology, Shengli Clinical Medical College of Fujian Medical University, Fuzhou, China; ^3^ Department of General Surgery, The Second Affiliated Hospital of Fujian Medical University, Quanzhou, China

**Keywords:** pancreatic adenocarcinoma, prognostic signature, DNA repair-related gene, chemotherapy, oxaliplatin

## Abstract

**Background:**

Pancreatic adenocarcinoma (PAAD) is one of the leading causes of cancer death worldwide. Alterations in DNA repair-related genes (DRGs) are observed in a variety of cancers and have been shown to affect the development and treatment of cancers. The aim of this study was to develop a DRG-related signature for predicting prognosis and therapeutic response in PAAD.

**Methods:**

We constructed a DRG signature using least absolute shrinkage and selection operator (LASSO) Cox regression analysis in the TCGA training set. GEO datasets were used as the validation set. A predictive nomogram was constructed based on multivariate Cox regression. Calibration curve and decision curve analysis (DCA) were applied to validate the performance of the nomogram. The CIBERSORT and ssGSEA algorithms were utilized to explore the relationship between the prognostic signature and immune cell infiltration. The pRRophetic algorithm was used to estimate sensitivity to chemotherapeutic agents. The CellMiner database and PAAD cell lines were used to investigate the relationship between DRG expression and therapeutic response.

**Results:**

We developed a DRG signature consisting of three DRGs (RECQL, POLQ, and RAD17) that can predict prognosis in PAAD patients. A prognostic nomogram combining the risk score and clinical factors was developed for prognostic prediction. The DCA curve and the calibration curve demonstrated that the nomogram has a higher net benefit than the risk score and TNM staging system. Immune infiltration analysis demonstrated that the risk score was positively correlated with the proportions of activated NK cells and monocytes. Drug sensitivity analysis indicated that the signature has potential predictive value for chemotherapy. Analyses utilizing the CellMiner database showed that RAD17 expression is correlated with oxaliplatin. The dynamic changes in three DRGs in response to oxaliplatin were examined by RT-qPCR, and the results show that RAD17 is upregulated in response to oxaliplatin in PAAD cell lines.

**Conclusion:**

We constructed and validated a novel DRG signature for prediction of the prognosis and drug sensitivity of patients with PAAD. Our study provides a theoretical basis for further unraveling the molecular pathogenesis of PAAD and helps clinicians tailor systemic therapies within the framework of individualized treatment.

## Introduction

Pancreatic adenocarcinoma (PAAD) is one of the most lethal malignancies; it has an increasing incidence worldwide, a poor prognosis, and a 5-year survival rate of less than 9% for all stages (1). Most patients are diagnosed at an advanced stage, and the average survival time is less than 12 months for patients diagnosed at any stage (2). The extremely high mortality rate and the dismal prognosis of PAAD patients are due to a lack of clinical symptoms at the early stages of the disease, a high recurrence rate, and high resistance to treatment, including chemotherapy and radiotherapy (3). Despite considerable advances in our understanding of the molecular basis of PAAD, little therapeutic progress has been made in this disease, in contrast to the progress that has been made in the treatment of various other cancers in recent decades (4). During that period, numerous clinical trials of treatments for PAAD have failed, and treatment options remain limited (5). The molecular and functional heterogeneity of PAAD may partially explain the lack of progress in clinical treatment (6). Unfortunately, our knowledge of the biological diversity of PAAD and of the genomic aberrations associated with it has not yet been translated into meaningful endpoints in patient survival. Thus, it is necessary to investigate the potential molecular mechanisms of PAAD and to develop effective prognostic models through which clinicians can assess the prognosis of patients.

The DNA damage response is a response that is commonly triggered by various exogenous and endogenous DNA lesions; its function is to maintain genomic integrity. A defective DNA damage response is an important hallmark of cancer (7). Defects in DNA damage repair pathways leading to the accumulation of genomic defects are closely linked to the initiation and progression of malignancies. In addition, deregulation of DNA repair pathways is associated with resistance to chemotherapy and radiotherapy (8). Based on genetic and molecular profiling, PAAD has been divided into several molecular subtypes (6). Molecular analysis has indicated that genetic alterations affect multiple signaling pathways in PAAD and that the DNA repair pathway is one of the key pathways involved (9). Several studies have reported that gene mutations, including those in BRCA1, BRCA2, TP53, MLH1, and CDKN2A, occur frequently in both sporadic and inherited PAAD (10, 11). Recently, novel therapeutic approaches that target DNA damage response pathways have been used to improve the response of tumors to chemotherapy (12, 13). Because of the importance of the DNA damage response in PAAD, approaches that target this biological process may provide therapeutic options that improve PAAD prognosis.

The development of next-generation sequencing (NGS) technology has led to its clinical application in the personalized treatment of cancer (14, 15). Recently, studies of prognostic biomarkers and treatment options for cancer patients have increasingly focused on multigene panels (16, 17). Multigene prognostic signatures could provide better accuracy in predicting cancer prognosis and could lead to more effective individualized treatment than the use of methods based on single genes and noncoding prognostic genes (18, 19). Because DNA damage is known to play a crucial role in cancer initiation, the use of multiple DNA repair-related genes (DRGs) to evaluate patient prognosis has attracted attention in the oncology field (20–22). However, systematic studies of prognostic and predictive biomarkers based on DNA repair genes in PAAD are lacking. Therefore, establishment of a DRG signature that can be used to predict disease prognosis and treatment responses in patients with PAAD has great clinical value.

The aim of the present study was to comprehensively evaluate the relationships between DRGs and PAAD. In our study, we developed a DRG signature based on DRGs that can accurately predict the prognosis of patients with PAAD and then constructed a predictive nomogram that is suitable for clinical application in PAAD patients. Furthermore, we analyzed the association between the DRG signature and individuals’ responses to chemotherapeutic drugs that are used in the treatment of PAAD. Our study provides a theoretical and practical basis for predicting the prognosis of PAAD patients and can help clinicians select effective treatment for patients based on risk assessment of their DRG signatures.

## Materials and methods

### Data acquisition and processing

We extracted RNA expression data in FPKM format and obtained clinical information on patients with PAAD from the TCGA database (https://portal.gdc.cancer.gov/). Only patients with complete survival data were included. A total of 172 patients from the TCGA database were used as a training set. Representative Gene Expression Omnibus (GEO) datasets containing more than 100 samples from PAAD patients, including RNA expression data and clinical information, were retrieved from the NCBI. The GSE71729 (*n* = 123), GSE21501 (*n* = 102), and GSE26939 (*n* = 115) datasets were downloaded from the GEO database (https://www.ncbi.nlm.nih.gov/geo/). A total of 340 patients from three GEO datasets were used as a validation dataset. We obtained a list of 276 human DNA repair genes from published resources (23–25). A total of 211 of 276 genes encompassed nine main DNA damage repair pathways, and 65 of 276 genes were associated with more than one DNA damage repair pathway or with coordinated molecular and cellular responses to DNA damage.

### Construction of the DRG signature

To assess prognosis-related DRGs in DNA repair gene sets, univariate Cox regression was used to calculate the correlation between the expression of each DRG and patient overall survival (OS) (*p*-value < 0.05). Next, least absolute shrinkage and selection operator (LASSO) Cox regression was applied to construct a DRG-related prognostic signature for PAAD patients. Finally, we obtained a risk scoring system that predicts the survival of patients. The following formula was used: risk score = (Expression gene 1 × Coefficient gene 1) + (Expression gene 2 × Coefficient gene 2) + · · · + (Expression gene *n* × Coefficient gene *n*). The patients in the training dataset were divided into high-risk and low-risk groups according to the median value of the risk score. The GSE71729 dataset, which contained 123 PAAD patients, was used to validate the DRG model. Kaplan−Meier analysis was performed to estimate the survival rates of the patients in the high-risk and low-risk groups. Time-dependent receiver operating characteristic (ROC) curves were plotted, and the area under the curve (AUC) for 1-year, 3-year, and 5-year OS in the training and validation datasets was calculated to evaluate the prognostic accuracy of the model.

### Development of a predictive nomogram

To explore the independent prognostic value of the risk score in PAAD patients, the patients’ clinical variables, including tumor grade, N stage, and age, were analyzed by univariate and multivariate Cox regression. Independent prognostic variables that were highly associated with OS were screened using univariate and stepwise multivariate Cox regression. We then integrated the risk score with independent prognostic variables using the R package “RMS” and constructed a predictive nomogram that predicts the survival probability of PAAD patients. The corresponding calibration curves were then graphically assessed to test the predictive accuracy of the nomogram. The discrimination ability of the nomogram was assessed using Harrell’s C-index (1,000 bootstrap resamples). The clinical utility of the prognostic nomogram was evaluated by decision curve analysis (DCA) (26).

### Gene set variation analysis and gene set enrichment analysis

To further analyze the biological processes in the low-risk and high-risk groups, we applied the “GSVA” R package for gene set variation analysis (GSVA) and enrichment analysis to unsupervised estimate pathway activity variations among certain populations. The gene set “c2.cp.kegg.v7.2. symbols” was downloaded from the MSigDB database and used to run GSVA. Clustering analysis was performed according to the pathway enrichment score for each patient. *p* < 0.05 and false discovery rate (FDR) <0.05 were considered statistically significant. Gene set enrichment analysis (GSEA) was performed to identify the biological processes (BP), molecular functions (MF), and cellular components (CC) in the high- and low-risk groups.

### Estimation of immune infiltration

The CIBERSORT algorithm with the gene signature matrix was used to quantify the proportions of 22 types of immune cells (27). RNA-Seq of PAAD samples was performed to obtain the abundance ratio matrix of 22 types of immune cells in each sample, including macrophages (M1 macrophages, M2 macrophages, and M0 macrophages), T cells (T follicular helper cells, resting memory CD4 T cells, activated memory CD4 T cells, γδ T cells, CD8 T cells, Tregs, and naïve CD4 T cells), resting natural killer (NK) cells, activated NK cells, resting mast cells, activated mast cells, memory B cells, resting dendritic cells (DC), activated DC, naïve B cells, monocytes, plasma cells, neutrophils, and eosinophils (28). CIBERSORT results for which *p* < 0.05 indicated that the inferred fractions of the immune cell populations produced by CIBERSORT were eligible for further analysis. The CIBERSORT output estimates were normalized, and immune cell-type fractions were summed to one. Single-sample gene set enrichment analysis (ssGSEA) was performed based on the expression of 29 immunity-related signatures and used to calculate enrichment scores for each sample using the “GSEAbase” R package (29).

### Estimation of individual sensitivity to chemotherapeutic agents and targeted agents

To evaluate the accuracy of the model in predicting the clinical response to chemotherapy and target therapy, the R package pRRophetic was used to calculate the half-maximal inhibitory concentration (IC_50_) of samples in the low- and high-risk groups by ridge regression (30). According to clinical practice guidelines, chemotherapeutic and targeted agents such as gemcitabine, oxaliplatin, docetaxel, paclitaxel, PARP inhibitors, and mTOR inhibitors were selected as candidate agents. Comparison of the IC_50_ values of the low- and high-risk groups was subsequently performed using the Wilcoxon signed-rank test. Drug sensitivity data were downloaded from the CellMiner database (https://discover.nci.nih.gov/cellminer/home.do) (31). We selected FDA-approved drugs and drugs that are currently undergoing clinical trials for analysis.

### Cell culture

PANC-1 and MIA PaCa-2 human PAAD cell lines were kindly provided by Stem Cell Bank, Chinese Academy of Sciences (Shanghai, China). PANC-1 and MIA PaCa-2 cell lines were cultured in RPMI-1640 and DMEM supplemented with 10% FBS and 1% penicillin/streptomycin. All PAAD cell lines were confirmed to be free of mycoplasma before use.

### Quantitative real-time polymerase chain reaction

PANC-1 (0.5 × 10^6^ cells/well) and MIA PaCa-2 (1 × 10^6^ cells/well) cells were seeded overnight in 60-mm plates. On the following day, PANC-1 and MIA PaCa-2 cells were treated with 25/50 μM oxaliplatin or DMSO for 24 h. The cells were then harvested, and their total RNA was extracted. The concentration of harvested RNA was measured using a NanoDrop 2000 spectrophotometer (ThermoFisher, USA). Total RNA was reverse-transcribed into cDNA using a Transcriptor First Strand cDNA Synthesis Kit (Roche, Germany). Quantitative real-time PCR (RT-qPCR) was performed using the SYBR Prime Script RT-PCR Kit (Invitrogen, USA). The primer sequences are listed in **Table S1**. All data were derived from three independent experiments. The Ct values were normalized to the genomic mean of GAPDH, the internal control. The relative expression levels were calculated using the 2^−ΔΔCT^ method. All results are presented as fold change relative to the internal control genes.

### Apoptosis assay

PANC-1 and MIA PaCa-2 cells were treated with 100 μM oxaliplatin for 24 h and then stained with Annexin V-PE and 7-AAD (BD Biosciences, USA) according to the manufacturer’s instructions. The percentages of apoptotic tumor cells were quantified by flow cytometry. Late apoptosis was defined as positive staining for both Annexin V and 7-AAD, and early apoptosis was defined as Annexin V-positive and 7-AAD-negative staining.

## Results

### Identification of prognosis-related DRGs and construction of the DRG signature

**Figure 1:**
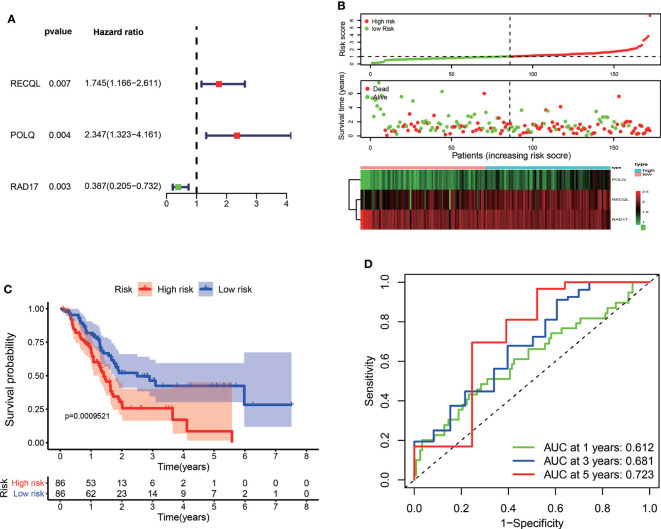
Construction of a prognostic signature for PAAD patients based on three DRGs. **(A)** Forest plot of the multivariate Cox regression analysis in the TCGA dataset. The red dots represented high-risk genes, while the green dots indicated low-risk genes. **(B)** The distribution of the risk scores, survival status, and expression profile of three DRGs between high-risk and low-risk groups. **(C)** Kaplan–Meier survival curves of OS between high-risk and low-risk groups. **(D)** The time-dependent ROC for 1-year, 3-year, and 5-year OS predictions for the DRG signature in the TCGA dataset.

In the training set, a total of 172 patients diagnosed with PAAD in the TCGA dataset were enrolled for development of the DRG signature. Univariate Cox regression analysis demonstrated that 82 DRGs were statistically significantly associated with the OS of PAAD patients (*p* < 0.05). Among them, 53 genes were risk factors (HR > 1), and 29 genes were protective factors (HR < 1) for PAAD patient prognosis. Consequently, LASSO Cox regression was conducted to screen the optimal prognosis-related DRGs and construct the DRG prognostic signature. Finally, three DRGs were selected and used to establish the prognostic signature (**Figure 1A**). The formula used to calculate the risk score was as follows: (0.42733 × RECQL expression) + (0.74947 × POLQ expression) + (−0.6574 × RAD17 expression). According to the median value of the risk score, pancreatic cancer patients were divided into high-risk and low-risk groups. The number of patients who experienced death increased as the risk score increased. The risk score distribution, OS, and the expression profiles of the three DRGs are shown in **Figure 1B**. Kaplan−Meier survival analysis indicated that the prognosis of patients in the low-risk group was better than that of patients in the high-risk group (**Figure 1C**). We used time-dependent ROC curves to evaluate the accuracy of the risk score. The AUC values for 1-year, 3-year, and 5-year OS were 0.612, 0.681, and 0.723, respectively (**Figure 1D**).

### Validation of DRG signature in an independent GEO dataset

**Figure 2:**
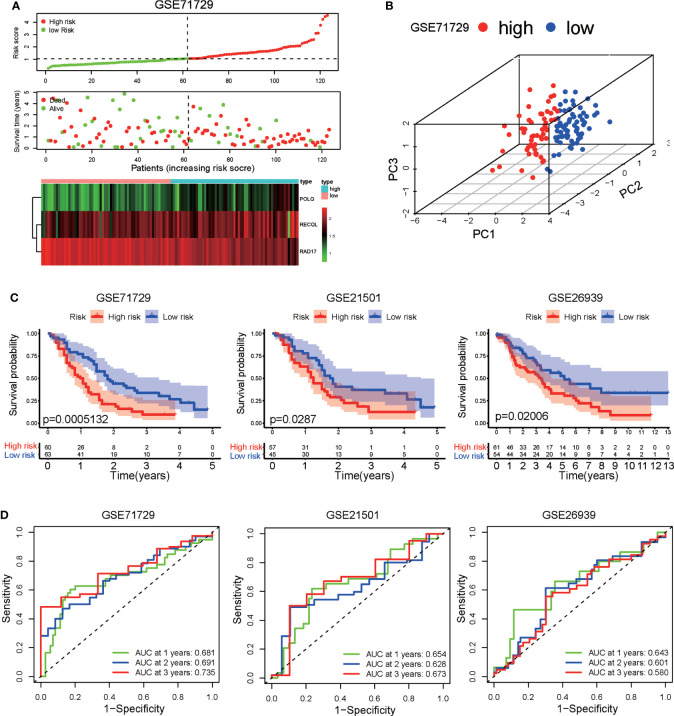
Validation of the DRG signature in three independent GEO datasets (GSE71729, GSE21501, and GSE26939). **(A)** Distribution of risk scores, survival status, and expression level of three DRGs in the GSE71729 dataset. **(B)** Principal component analysis based on the expression levels of three DRGs in the GSE71729 dataset. **(C)** Kaplan−Meier analysis of OS in the high-risk and low-risk groups in three GEO datasets. **(D)** Time-dependent ROCs for 1-year, 2-year, and 3-year OS predictions based on the DRG signature in three GEO datasets.

To validate the predictive capability of the DRG signature, the GSE71729, GSE21501, and GSE26939 datasets were used as external validation datasets. Risk scores were calculated for the PAAD patients in the validation dataset using the formula presented above, and the patients in the validation dataset were then divided into high-risk and low-risk groups according to the median value of the risk score in the training set. The distributions of risk score, patient status, and gene expression of the three DRGs for the patients in the validation dataset are shown in **Figure 2A**. Principal component analysis (PCA) showed that the PAAD patients in the GSE71729 validation dataset could be clearly categorized into two groups based on the prognostic signature (**Figure 2B**). Consistent with the findings obtained for the training set, the survival rate of the PAAD patients in the high-risk group was lower than that of the patients in the low-risk group in all three external validation datasets (**Figure 2C**). The AUCs of the ROC curves showed that the DRG signature had good predictive performance for PAAD patients in these external validation datasets (**Figure 2D**). Taken together, these results indicate that the DRG prognostic signature has great external validity and reliability for PAAD patients. We compared the prognostic performance of the DRG signature with the performances of other multigene prognostic signatures in PAAD. The C-index and RMS curve showed that the prognostic performance of the DRG signature was similar to the performances of the four signatures described in previous studies (32–35) (**Supplementary Figures S1A, B**).

### Construction of a predictive nomogram based on the DRG signature

**Figure 3:**
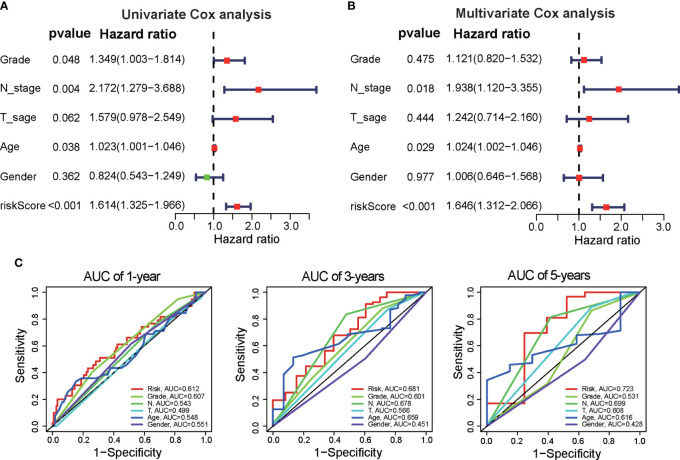
Independence of the DRG signature for survival prediction. **(A)** Univariate Cox regression analysis was performed to validate the prognostic values of clinical factors and risk scores in the TCGA dataset. **(B)** Multivariate Cox regression analysis was performed to verify the prognostic values of clinical factors and risk scores. **(C)** Time-dependent ROC analysis of the performance of the DRG signature in predicting clinical factors at 1 year, 3 years, and 5 years.

We further assessed the independence of the prognostic signature in predicting the prognosis of PAAD patients. Univariate Cox regression analysis demonstrated that clinical variables, including tumor grade, N stage, age, and risk score, were associated with patient prognosis (**Figure 3A**). Multivariate Cox regression analysis revealed that N stage, age, and risk score were independent indicators correlated with OS (**Figure 3B**). The ROC curves showed that the DRG signature had better prognostic performance for 1-year, 3-year, and 5-year OS than did age, tumor grade, and N stage (**Figure 3C**). These results suggest that the DRG signature could be used as an independent predictor in clinical application.

**Figure 4:**
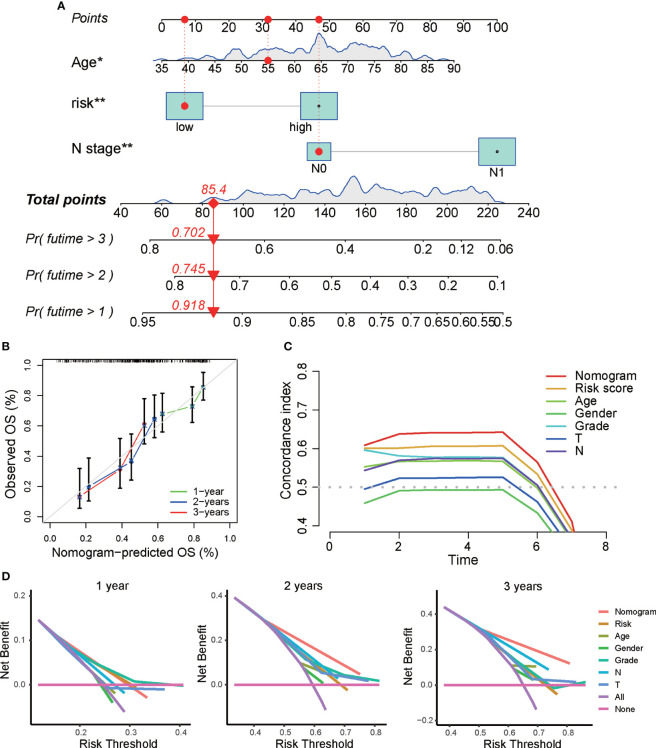
Construction of a predictive nomogram based on the DRG signature. **(A)** Nomogram based on the prognostic signature consisting of risk score and clinical factors. **(B)** Calibration plot showing the predictive accuracy of the nomogram at 1 year, 3 years, and 5 years. **(C)** C-index of the prognostic nomograms, risk score, and other clinical factors. **(D)** DCA curve for the prediction of 1-year, 3-year, and 5-year overall survival. *p < 0.05, **p < 0.01, ***p < 0.001.

Next, we constructed a prognostic nomogram to predict 1-year, 3-year, and 5-year OS using the DRG signature and the independent clinical factors we identified above (**Figure 4A**). The calibration curve demonstrated optimal correspondence between the predicted and actual values for 1-year, 3-year, and 5-year survival probabilities, indicating that the nomogram has good ability to distinguish most survival outcomes at these time points (**Figure 4B**). The C-index of the prognostic nomograms was higher than that of the risk score and those of other clinical variables, indicating that it has better discrimination ability than those indicators (**Figure 4C**). Furthermore, DCA was applied to evaluate the clinical utility of the prognostic nomogram. The results demonstrated that the nomogram yields a higher net benefit than does the TNM staging system (**Figure 4D**). Taken together, these results demonstrate that the predictive nomogram is a valuable indicator for prediction of the prognoses of patients with PAAD.

### Clinical correlation and functional evaluation of the DRG signature

**Figure 5:**
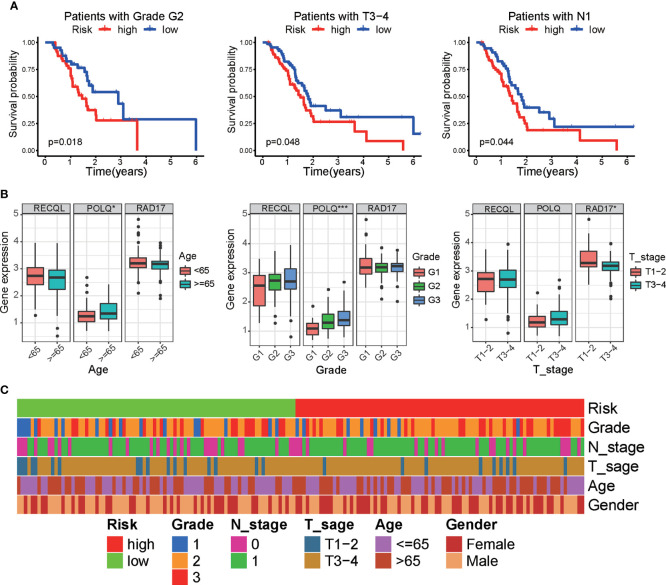
Correlation between clinical parameters and the DRG signature. **(A)** Kaplan−Meier survival analysis of the overall survival of patients between the prognostic signature and different clinical subgroups. **(B)** Boxplot showing the correlation between three DRGs and clinical characteristics in PAAD patients. **(C)** Heatmap showing the associations between risk score and clinicopathological characteristics. *p < 0.05, ***p < 0.001.

We next investigated the prognostic efficacy of the prognostic signature in different clinical subgroups of PAAD patients. Kaplan−Meier survival analysis showed that in patients with PAAD of grade G2, T3-4 and N1 pathologic stages, the survival time of the low-risk group was longer than that of the high-risk subgroup (**Figure 5A**). In addition, we performed clinical correlation analysis to investigate the correlation between three DRGs, risk score, and clinical characteristics in PAAD patients. The results of the correlation analysis demonstrated that POLQ expression was increased in older patients (≥65 years of age) and in tumors of higher grade, while RAD17 expression was higher in tumors of low clinical T stage (**Figure 5B**). Analysis of the association between the risk scores and the patients’ clinical characteristics showed that there were no significant correlations between risk score and clinical characteristics (**Figure 5C** and **Supplementary Figure S2A**).

**Figure 6:**
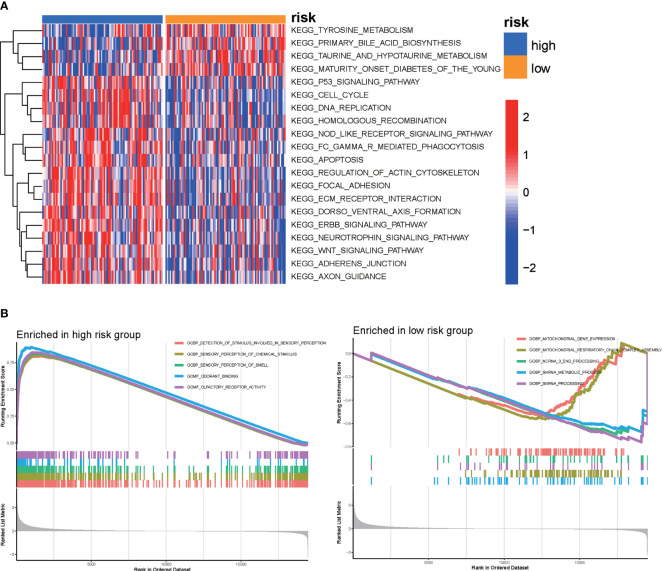
GSVA was used to perform functional annotation between the high-risk and low-risk groups of PAAD patients. **(A)** Heatmap of GSVA between the high-risk and low-risk groups. **(B)** GSEA of biological functions between the high-risk and low-risk groups.

According to the MSigDB hallmark gene sets, we used GSVA to investigate the KEGG pathways associated with the DRG signature. Our results, which are shown in the heatmap, demonstrated that the high-risk group was enriched in tyrosine metabolism, primary bile acid biosynthesis, and taurine and hypo-taurine metabolism. The p53 signaling pathway, the cell cycle, DNA replication, and the homologous recombination pathway were enriched in the low-risk group (**Figure 6A** and **Supplementary Figure S2B**). In addition, GSEA was applied to identify the biological functions enriched in the high-risk and low-risk groups. The GSEA results indicated that the high-risk group was enriched in protein complex assembly, sister chromatid segregation, and the histone deacetylase histone pathway, while the low-risk group was enriched in detection of stimuli involved in sensory perception, sensory perception of chemical stimuli, and the dendrite membrane pathway (**Figure 6B**).

### The landscape of tumor microenvironment immune cell infiltration in the two risk groups

**Figure 7:**
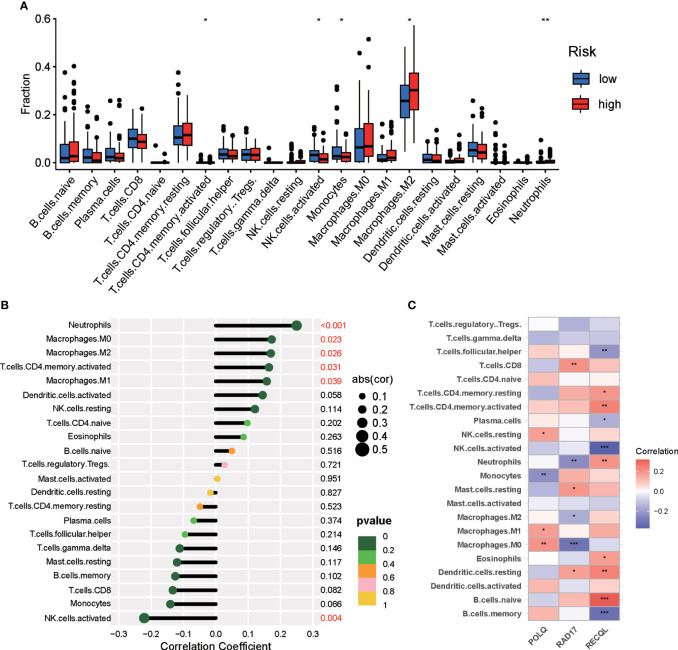
Differences in immune infiltration between the two risk groups of PAAD patients. **(A)** Violin plot demonstrating the ratio differentiation between the high-risk and low-risk groups for 22 types of tumor-infiltrating immune cells. **(B)** Correlation analyses between risk score and tumor-infiltrating immune cells in PAAD patients. **(C)** Correlation analyses for the expression of three DRGs and tumor immune cells. *p < 0.05, **p < 0.01, ***p < 0.001.

Increasing evidence has indicated that DNA repair may affect immune cell infiltration of the tumor microenvironment. To explore the association between the DRG signature and the level of tumor infiltration by immune cells in pancreatic tumors, the CIBERSORT algorithm was used to calculate the proportions of tumor-infiltrating immune cells in each PAAD patient. The differential analysis results demonstrated that the proportions of activated NK cells and monocytes were higher in the low-risk group, while M2 macrophages, activated CD4 memory T cells, and neutrophils were increased in the high-risk group (**Figure 7A**). Correlation analyses of tumor-infiltrating immune cells revealed that activated NK cells were negatively correlated with risk score and that M2 macrophages, activated CD4 memory T cells, and neutrophils were positively correlated with risk score (**Figure 7B**). We further investigated the correlation of the DRGs with tumor-infiltrating immune cells in PAAD patients. The correlations between the expression of the three DRGs and the number of immune cells within tumors are shown in **Figure 7C**.

**Figure 8:**
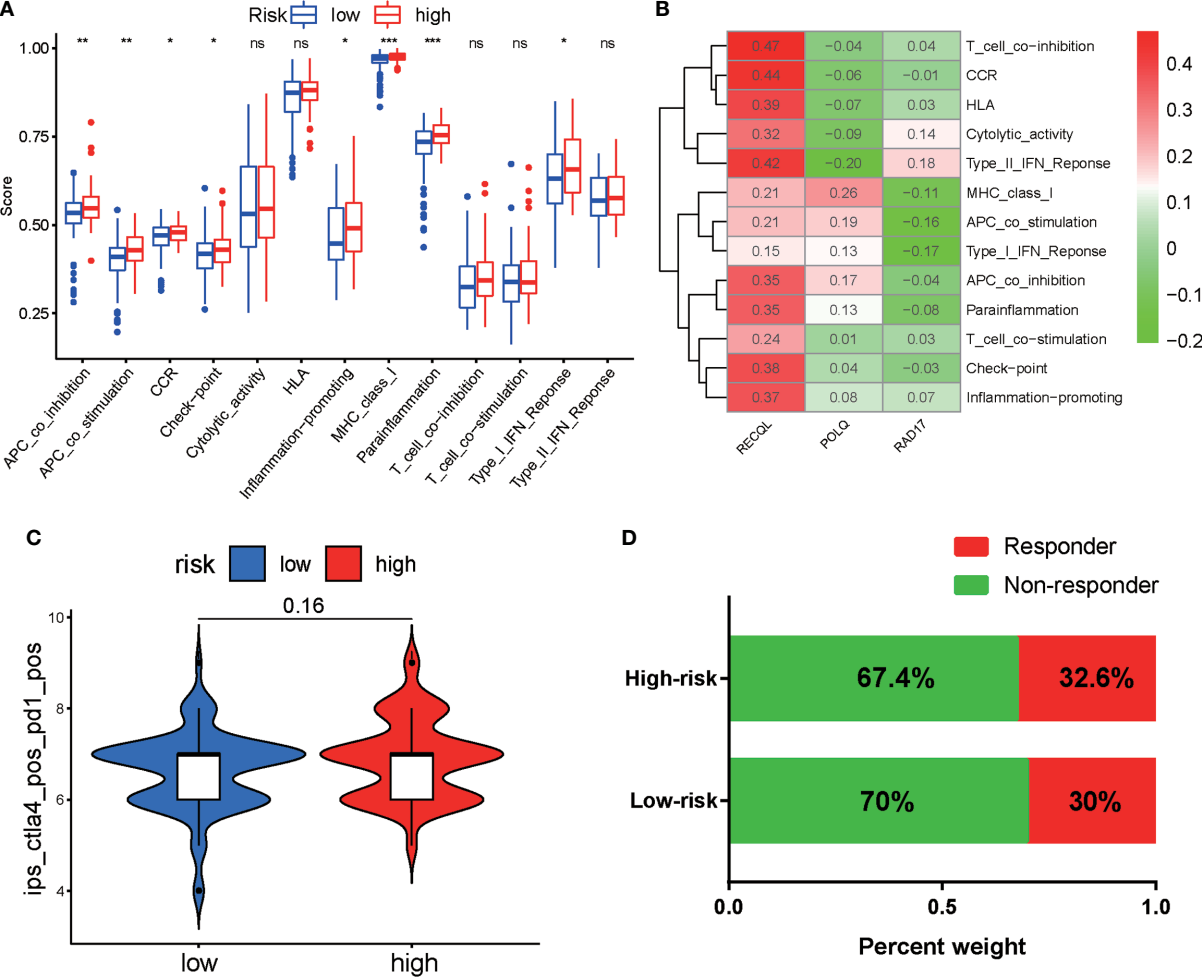
Correlation analyses and evaluation of immunotherapy sensitivity in the two risk groups of PAAD patients. **(A)** Correlation analyses of immune-related functions in the high-risk and low-risk groups of PAAD patients. **(B)** Correlation analyses of the expression of three DRGs and immune-related pathways. **(C)** Violin plot showing the IPS for CTLA-4 and PD-1 inhibitors between the two groups based on the TCIA database. **(D)** Comparison of the responses of the two groups to immunotherapy using the ImmuCellAI algorithm. *p < 0.05, **p < 0.01, ***p < 0.001. ns, not significant.

Next, we used the ssGSEA algorithm to calculate the immune-related functions of PAAD patients in the high-risk and low-risk groups. The results indicated that the high-risk group was significantly linked to immune-related pathways, including APC coinhibition, APC costimulation, MHC class I, and the checkpoint pathway (**Figure 8A**). Correlation analyses demonstrated that RECOL was positively associated with most immune-related pathways, while RAD17 was negatively correlated with most immune-related pathways (**Figure 8B**). Furthermore, we evaluated the correlation between risk score and the expression of inhibitory checkpoint molecules (PD-1, PD-L1, PD-L2, CTLA-4, TIM-3, LAG-3, VISTA, and others). We found that expression of PD-L1 (CD274), PD-L2 (PDCD1LG2), and TIGIT was upregulated in the high-risk group (**Supplementary Figure S3A**). Finally, we evaluated the potential response of each patient to immunotherapy using the TCIA and ImmuCellAI algorithms. The relative probability of responding to anti-PD-1/PD-L1 and anti-CTLA-4 treatment was similar in the two risk groups, according to the IPS values of the TCIA predictive model (**Figure 8C**) and the ImmuCellAI algorithm analysis (**Figure 8D**).

### Evaluation of the sensitivities of the patients in the two risk groups to chemotherapeutic drugs

**Figure 9:**
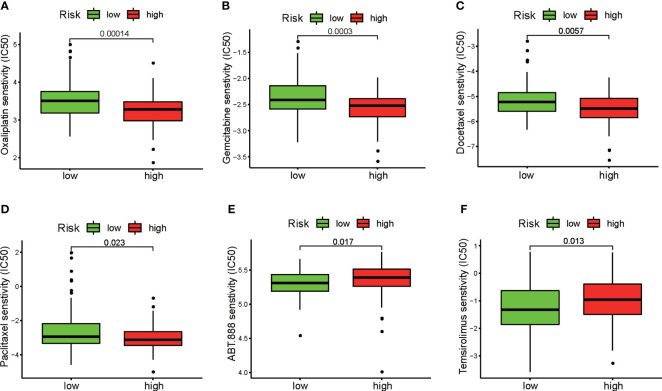
Evaluation of sensitivity to chemotherapeutic drugs and target therapeutic agents in the two risk groups. Differences between the high-risk and low-risk groups in response to chemotherapy based on IC_50_ values for **(A)** oxaliplatin, **(B)** gemcitabine, **(C)** docetaxel, and **(D)** paclitaxel. Differences between the high-risk and low-risk groups in response to targeted therapy based on IC_50_ values for **(E)** the PARP inhibitor ABT.888 and **(F)** the mTOR inhibitor temsirolimus.

We next evaluated the association between the patients’ risk scores and their clinical responses to chemotherapy and targeted therapy for PAAD. The pRRophetic algorithm was used to predict the responses of the patients in the high-risk and low-risk groups to several first-line chemotherapeutic drugs and target agents. Our study revealed that the IC_50_ values for oxaliplatin (**Figure 9A**), gemcitabine (**Figure 9B**), paclitaxel (**Figure 9C**), and docetaxel (**Figure 9D**) were significantly higher in samples from patients in the low-risk group than in samples from patients in the high-risk group. Interestingly, the low-risk group demonstrated much higher sensitivity to the PARP inhibitor ABT.888 (**Figure 9E**) and the mTOR inhibitor temsirolimus (**Figure 9F**) than did the high-risk group. These results indicate that the DRG signature has potential predictive value for chemosensitivity and targeted therapy.

### Validation of three prognostic genes following chemotherapy

**Figure 10:**
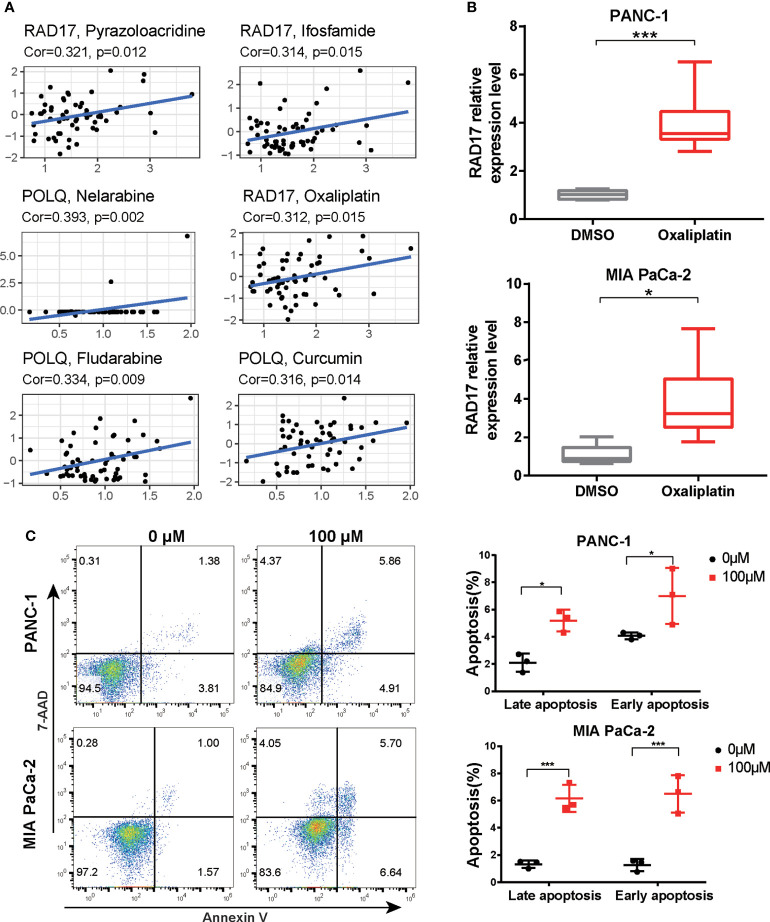
Expression levels of three DRGs in response to oxaliplatin treatment. **(A)** Correlation scatter plot showing the relationship between the IC_50_ value of the drug in the CellMiner database and the expression levels of three DRGs in the signature. Six groups of relationships were selected. **(B)** PANC-1 and MIA PaCa-2 cells were treated with oxaliplatin for 24 h. The expression of RAD17 was measured by RT-qPCR. **(C)** PANC-1 and MIA PaCa-2 cells were treated with 100 μM oxaliplatin. The percentages of early and late apoptotic cells were determined and are shown in the right panel. *p < 0.05, ***p < 0.001.

We then investigated the association between the three DRGs in our signature and the IC_50_ values for 263 FDA-approved drugs and clinical trial drugs from the CellMiner database. Our results showed that pyrazoloacridine, ifosfamide, and oxaliplatin were significantly correlated with RAD17 expression and that POLQ expression was associated with nelarabine, fludarabine, and curcumin (**Figure 10A**). Oxaliplatin was the most common drug used to treat PAAD. We further validated the expression levels of the three prognostic DRGs in response to chemotherapeutic drugs in PAAD cell lines. The expression of the three DRGs after oxaliplatin treatment of two PAAD cell lines was examined by RT-qPCR. The results of RT-qPCR showed that treatment with oxaliplatin resulted in upregulation of RAD17 expression in both the PANC-1 and MIA PaCa-2 cell lines (**Figures 10B, C**). However, there was no significant difference in RECQL or POLQ expression after oxaliplatin treatment of the PAAD cell lines (**Supplementary Figures S3B, C**). Cell apoptosis was also measured by flow cytometry after treatment with oxaliplatin. We found that oxaliplatin induced early and late apoptosis in PAAD cells (**Figure 10D**). These results suggest that overexpression of RAD17 may confer resistance to chemotherapy in PAAD and that this resistance may be associated with modulation of apoptosis.

## Discussion

DNA damage repair pathways are triggered to conserve genomic integrity and stability when cells are exposed to various types of DNA lesions (36). Unrepaired DNA lesions induce the accumulation of numerous genetic changes that can lead to the development of a variety of cancers and other diseases (37). Therefore, DNA damage repair pathways are regarded as attractive tumor therapeutic targets. DRGs are involved in repairing various types of DNA damage, and this could broaden treatment options for patients. In our study, we systematically analyzed the prognostic performance of DRGs for predicting OS in PAAD and developed a novel signature that includes RECQL, POLQ, and RAD17. This study provides a theoretical foundation for prediction of prognosis and selection of treatment strategy for patients with PAAD.

In recent years, dysfunctional DNA damage repair has been increasingly recognized as a new hallmark of PAAD (38). Approximately 20% of PAAD patients have germline and somatic mutations in genes involved in DNA damage repair, such as BRCA1, BRCA2, MSH2, MSH6, MLH1, FANCF, and PALB2, all of which are involved in homologous recombination repair or mismatch repair (39). Mutations in genes that encode proteins involved in DNA damage repair pathways create a highly heterogeneous mutational background that gives each patient a unique molecular cancer signature. Nicola et al. classified PAAD into four subtypes: stable, locally rearranged, scattered, and unstable, according to the observed genomic alterations. Unstable PAAD patients with high BRCA mutational signatures have shown sensitivity to platinum-based chemotherapy (40). Amikar et al. found that the presence of DRG mutations, including germline BRCA1/2 mutations, was associated with longer OS in PAAD patients treated with chemotherapy (41). In our study, we constructed a DRG model based on three prognostic DRGs using univariate analysis and multivariable analyses to predict the prognosis of patients with PAAD. Moreover, independent prognostic analysis and verification of the DRG model based on the GEO dataset confirmed that the risk model effectively predicts the prognosis of PAAD patients. However, this study revealed that N stage is an independent indicator of OS, but T stage did not appear to be an independent indicator for pancreatic cancer. One reason for this may be that most patients are diagnosed at an advanced stage of the disease or may even have metastasis at diagnosis. The prognosis for patients with pancreatic cancer is so poor that the effect of T stage is less relevant once metastasis exists.

Of the genes in the three-gene prognostic signature RECQL belongs to the RecQ DNA helicase family, the members of which are known to interact with several important factors required for DNA repair (42). Matsushita et al. showed that RECQL1 is a prognostic factor for ovarian cancer and that it contributes to potential malignancy by inhibiting apoptosis. Li et al. reported that RECQL variant genotypes are associated with cancer risk and with the clinical outcomes of PAAD patients (43). Our results demonstrated a negative correlation between RECQL gene expression and patient OS. However, no correlation with patient age, grade, or T stage was observed. POLQ, which encodes a DNA polymerase involved in DNA double-strand break repair, was found to be associated with risk of familial PAAD development (44). Previous research showed that overexpression of POLQ is linked to unfavorable prognosis in a variety of malignancies, a finding that is consistent with our results (45, 46). At present, POLQ inhibitors, which represent a valuable therapeutic strategy for a range of cancers associated with BRCA mutations, including PAAD, are in development for clinical use (47). Rad17 is an essential protein that recruits DNA damage response complexes to DNA. Xu et al. reported that depletion of RAD17 potentiates gemcitabine cytotoxicity in PAAD cells (48). Our results are consistent with the data showing that inhibition of RAD17 potentiates oxaliplatin cytotoxicity in PAAD cell lines. The synergistic effect of RAD17 knockdown can partially explain why the low-risk group was more sensitive to increased chemotherapy than the high-risk group.

Recent preclinical and clinical studies have revealed that the DNA damage response, which promotes the generation of tumor neoantigens, profoundly impacts multiple aspects of tumor immunogenicity and interactions with the tumor cell microenvironment (49). Recent clinical trials assessing immunotherapy have demonstrated the potential of DNA repair-related biomarkers to predict sensitivity to immunotherapy (50). Our analyses show that the stromal score correlates negatively with the risk score, and the expression of the coinhibitory checkpoint molecules PD-1, PD-L1, and TIGIT was increased in the high-risk score group compared with the low-risk score group. These results indicate that high stromal infiltration may be an unfavorable prognostic factor in PAAD. Whether higher expression of stromal infiltration and coinhibitory checkpoint molecules is associated with a better response to immunotherapy in the high-risk group requires further exploration. However, the relative probabilities of response to anti-PD-1/PD-L1 and anti-CTLA-4 treatment were similar in the two risk groups in our study. In addition, our results indicated that the higher levels of CD8 T-cell and activated NK-cell infiltration in the low-risk group might be correlated with the presence of an immunoactive tumor microenvironment, providing a possible explanation for the better prognosis and sensitivity to chemotherapy of these PAAD patients.

Cytotoxic chemotherapeutic agents, including gemcitabine, paclitaxel, fluorouracil, and oxaliplatin, remain the standard first-line treatment for unresectable PAAD and prolong the survival of patients with metastatic PAAD. However, the median OS in advanced-stage PAAD rarely exceeds 12 months (51). Recently, molecular markers have been employed to effectively select patients for therapy with specific anticancer agents (6). Oxaliplatin is a first-line drug in the FOLFIRINOX or GEMOX/XELOX regimen for PAAD patients. In our study, we found that RAD17 expression is significantly correlated with the oxaliplatin response. DNA damage repair is one of the factors responsible for oxaliplatin resistance (52). RAD17 plays a crucial role in responses to DNA damage and in double-strand break repair (53). The higher expression of critical DNA repair genes such as RAD17 suggested that patients in the low-risk group may have high DNA repair capacity, which could partially explain the higher IC_50_ values in the low-risk group. In addition, several clinical trials have produced evidence that PARP inhibitors have potent antitumor effects in BRCA-mutant PAAD (54), and these agents have been approved by the FDA for the treatment of metastatic PAAD in patients with BRCA mutations. Preclinical studies have also demonstrated that mTOR is a potential therapeutic target for PAAD (55). Our data showed that the high-risk group obtained greater therapeutic benefit from treatment with PARP inhibitors and mTOR inhibitors, indicating that the DRG signature is a novel biomarker for predicting the response to targeted therapy. PARP activity is essential for the repair of DNA breaks *via* the base excision repair pathway, which means inhibition of PARP1 could target DNA damage repair pathways and impair DNA damage repair ability. It is part of the reason why patients in the low-risk group showed higher sensitivity to the PARP inhibitor. In addition, we have compared the prognostic performance of the DRG’s signature with those of other multigene prognostic signatures in previous studies. Although DNA damage repair is widely involved in prognosis, chemosensitivity, and metastasis of PAAD and DNA damage repair pathways have been reported as a new target in PAAD, a DRG’s signature based on DRGs in PAAD has not been reported. Our DRG’s multigene signature may provide a theoretical basis for the research and additional targets for treatment.

This study has two major limitations. First, it is based on bioinformatics analysis of publicly available human PAAD datasets. Although the DRG signature has been validated in independent datasets, prediction of the response of patients with PAAD to chemotherapy has been a complex issue. Multicenter prospective trials are needed for further validation of the predictive model. The second major limitation of this study is that there is a lack of basic experimental research on the signaling pathways related to the three identified DRGs and on therapeutic responsiveness to agents such as toxaliplatin. We will continue to investigate the molecular mechanism underlying the relationship between the DRGs and therapeutic responsiveness in future research.

## Conclusion

In summary, we developed a novel DRG predictive model based on RECQL, POLQ, and RAD17 to predict the prognosis of patients with PAAD. A nomogram based on clinical factors and on the DRG signature was constructed for use as an individual clinical prediction tool. Further study of the DRG signature demonstrated that the prognosis risk signature can be applied as a potential predictive marker of response to treatment in patients with PAAD. Our study provides a theoretical basis for further unraveling the molecular pathogenesis of PAAD and helps clinicians tailor systemic therapies within the framework of individualized treatment.

## Data availability statement

The datasets presented in this study can be found in online repositories. The names of the repository/repositories and accession number(s) can be found in the article/**Supplementary Material**.

## Author contributions

JL and AZ planned and designed this study. JL and WC analyzed and acquired data. JH and AZ helped interpret the results. JL, JH and AZ drafted the manuscript. All authors revised and reviewed this work, and all authors gave their final approval of the submitted manuscript.

## Funding

This work was supported by the Natural Science Foundation of Fujian Province (Grant No. 2021J01247), the Doctoral Project of the Second Affiliated Hospital of Fujian Medical University (Grant No. BS202110) to JL, and the Science and Technology Project of Quanzhou City (Grant No. 2018N015S) to AZ.

## Conflict of interest

The authors declare that the research was conducted in the absence of any commercial or financial relationships that could be construed as a potential conflict of interest.

## Publisher’s note

All claims expressed in this article are solely those of the authors and do not necessarily represent those of their affiliated organizations, or those of the publisher, the editors and the reviewers. Any product that may be evaluated in this article, or claim that may be made by its manufacturer, is not guaranteed or endorsed by the publisher.
