# Supplementary material for: Determination of a DNA repair-related gene signature with potential implications for prognosis and therapeutic response in pancreatic adenocarcinoma
Source: Front Oncol. 2022 Oct 24;12:939891. doi: 10.3389/fonc.2022.939891 (PMC9638008; doi:10.3389/fonc.2022.939891)
Supplement: Supplementary file 1 [file DataSheet_1.docx]

**Supplementary Figures**


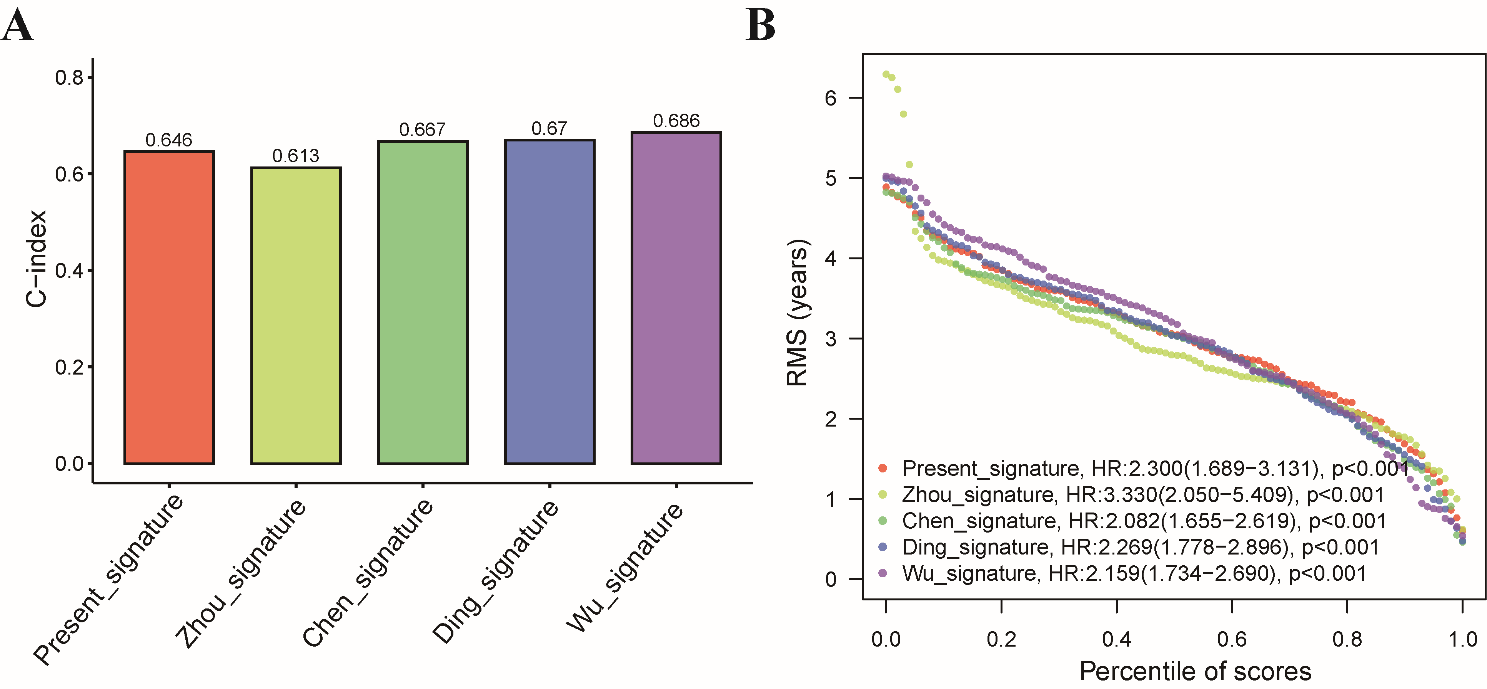


**Supplementary Figure S1 | (A)** The C-index for DRG’s signature and other 4 prognostic signatures. **(B)** The RMS time curves for DRG’s signature and other 4 prognostic signatures.


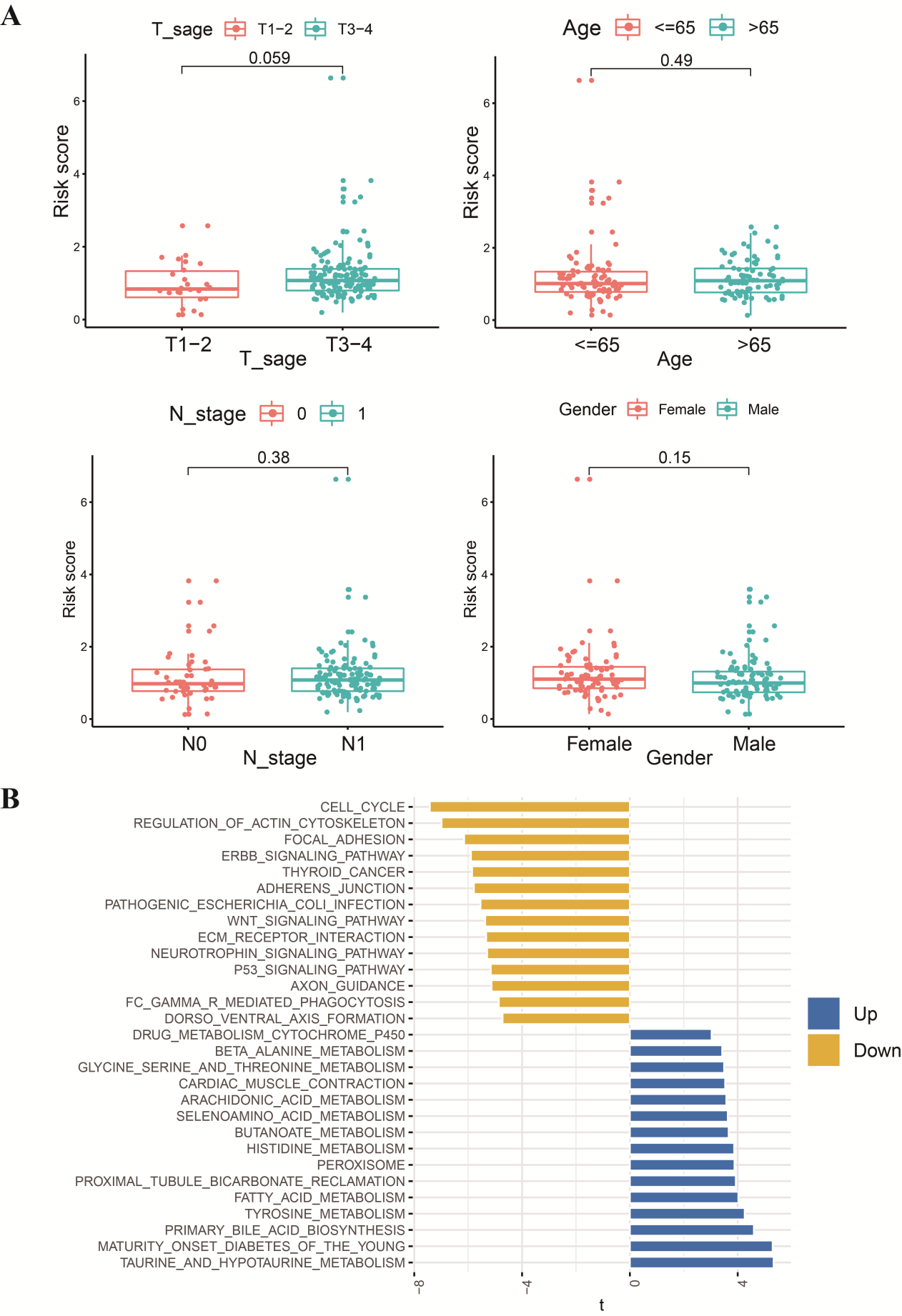


**Supplementary Figure S2 | (A)** The scatter plot showed the correlation between risk score and clinical characteristics (Age, gender, T Stage and N stage). **(B)** Barplot of “Hallmark” pathway score calculated by GSVA for two groups in PAAD patients.


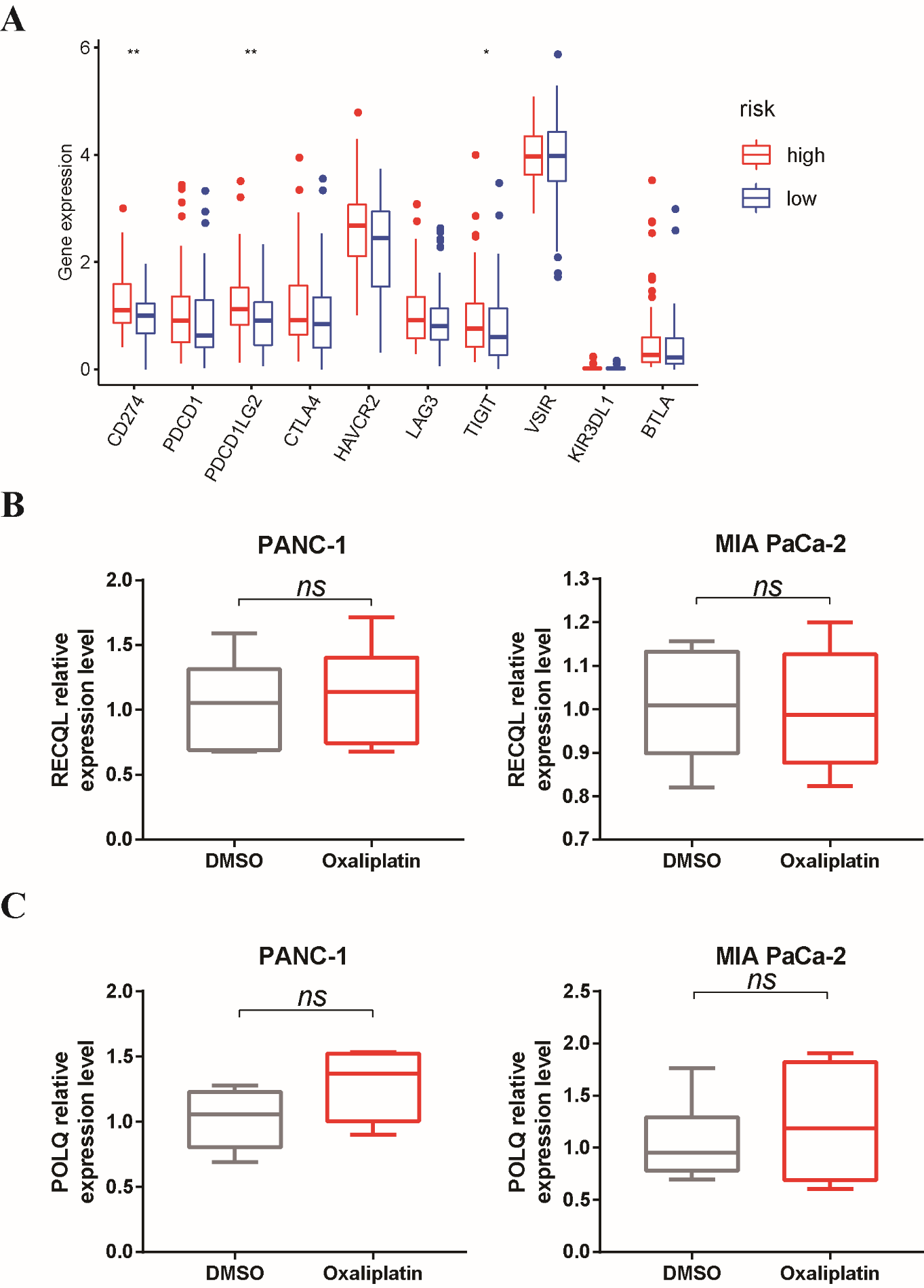


**Supplementary Figure S3 |** **(A)** The expression level of inhibitory checkpoint molecules including PD-1, PD-L1, PD-L2, CTLA-4, TIM-3, LAG-3, VISTA, TIGIT, KIR and BTLA between two risk groups in PAAD patients. **(B)** PANC-1 and MIA PaCa-2 cells were treated with oxaliplatin for 24 hours. The expression levels of RECQL and **(C)** POLQ cell lines were measured by RT-qPCR. * p<0.05, ** p<0.01, *** p<0.001.

**Table S1 | Sequences of the primer used for RT-qPCR**

| Genes | Forward primer | Reverse primer |
| --- | --- | --- |
| RAD17 | GGTCCAAGCTATTGGTGGCAAAG | AATGAGAGGGCAACCGAGGTGA |
| RECQL | GGAGCCAGAAGATAAGACCACAG | GGATAACAAACCTCACATCTGGC |
| POLQ | CTTGTGGCATCTCCTTGGAGCA | AATCCCTTGGCTGGTCTCCATC |
| GAPDH | GTCTCCTCTGACTTCAACAGCG | ACCACCCTGTTGCTGTAGCCAA |
